# Antioxidants and Prooxidants: Effects on Health and Aging

**DOI:** 10.1155/2018/1472708

**Published:** 2018-02-28

**Authors:** Márcio Carocho, Isabel C. F. R. Ferreira, Patricia Morales, Marina Soković

**Affiliations:** ^1^Mountain Research Centre (CIMO), Polytechnic Institute of Bragança, Bragança, Portugal; ^2^Complutense University of Madrid, Madrid, Spain; ^3^University of Belgrade, Belgrade, Serbia

Reactive species are compounds related to two types of molecules, the reactive oxygen species and the reactive nitrogen species. These compounds are produced in the normal metabolism of cells and may take part in the pathological process named oxidative stress, which is promoted when the balance between free radicals and antioxidants tends to favour the former. As a result, the excess of free radicals within the human body leads to oxidative stress, resulting in the development or potentiation of many types of diseases, namely, chronic disorders, cardiovascular problems, diabetes, cancer, and rheumatoid arthritis [1].

A few decades ago, the belief regarding free radicals and oxidative stress was that they were completely undesired and dangerous for the human body and needed to be removed from our bodies immediately after their formation, either through endogenous or exogenous (diet) antioxidants. Today, this demonizing view has mellowed down and it is known that oxidative stress is part of the innate human immune system, and that free radicals are also related to defence mechanisms, but also used as cell mediators, pathway signalling, cell differentiation, proliferation and migration inducers, and release or inactivate the endothelium-derived relaxing factor, among other processes [1, 2].

The aim of this special edition is to shed some light on the effects of antioxidants and prooxidants on diseases and their overall impact on aging, following the free radical theory of aging. The initial focus of aging was centred on the free radical damage to macromolecules; however, it has become clear that hydroperoxides like H_2_O_2_ play important roles in physiologic signalling. Thus, the dichotomy resides on the fact that alterations in redox signalling are observed in aging, but, on the other hand, deviation from redux homeostasis can result in disease, making the study of redox signalling a hot topic. Furthermore, the free radical theory of aging has been threatened after recent discoveries on the importance of hydroperoxides and other radicals in the human body. Many scientists claim that it is should not be considered a single theory, but a part of a wider general theory of aging [3, 4]. Despite the free radical theory of aging being questioned, the link between free radicals, oxidative stress, and many diseases is scientifically proven. Examples of these diseases are neurodegenerative ones, which, with increasing life expectancy will also increase in the coming decades. New and unusual methods are being tested for these types of diseases, namely, by fine-tuning antioxidant concentrations, exhaustive study of antioxidant mechanisms and interactions, modulation of oxidative stress, and other therapeutic approaches [5–7].

This special edition, comprised of 9 original research articles and 4 reviews on various aspects, namely, pro and antioxidant functions of the peroxisome-mitochondria connection and its impact on aging and disease, the impact of oxidative stress on the regeneration of pancreatic beta cells, the impact of metallothionein in brain disorders, and finally an extensive overview of the role of flavonoids as putative inducers of transcription factors Nrf2, FoxO, and PPAR*γ*. In terms of research manuscripts, four of these describe the role of specific plant compounds in the human body or in test subjects (mice and rats), namely, the cardioprotective effects of salvianic acid A and raloxifene in mice with elevated homocysteine and estrogen-deficient rats, respectively. The effects of berberine as preconditioning agent to reduce premature senescence of cells were also researched, as well as the effect of resveratrol on rats' cognitive performance with aging. Plant extracts were also described as having cardioprotective effects and reducing oxidative stress, while the *in vitro* antioxidant and antifibrotic profile of other herbal formulations was tested. Dietary supplements, commonly reported as being effective against many neurodegenerative diseases and having antioxidant activity, were also tested in terms of quality control by liquid chromatography with the aid of a mass spectrometer. Finally, using new agar diffusion methods, the risk of tumors was evaluated through the measurement of urinary and serumal antioxidants.

The editors hope that the published articles of this special edition may be of interest to all readers, and overall, be of aid to further improve the research on free radicals, antioxidants and prooxidants, aging, and related diseases.
